# Knowledge of inpatient diabetes among sixth-year medical students

**DOI:** 10.1186/1758-5996-7-S1-A186

**Published:** 2015-11-11

**Authors:** Silmara Aparecida de Oliveira Leita, Suelen do Carmo Vieira, M Cecilia Lansang

**Affiliations:** 1 Hospital Cruz Vermelha Brasileira, Curitiba, Brazil

## Background

The importance of proper management of inpatient hyperglycemia is increasingly being recognized. However, the curriculum for medical school has lagged behind current clinical recommendations.

## Objective

The aim of this study was to assess the knowledge of medical interns on inpatient diabetes.

## Materials and methods

The questionnaires were given on inpatient diabetes. It was the same previously applied in Lansang study with adaptations for the Brazilian reality. Descriptive analysis was used. The test was administered to 44 intern medical students, with an error margin of 5% and a 95% confidence level.

## Results

82% of students performed the initial management of acute coronary syndrome correctly. However, only 68% of them hit the proper management of diabetes in the same patient, 18% of students opted for the maintenance of oral antidiabetic during hospitalization. In another patient assessed with COPD only 23% of students indicated insulin properly, another 43% recommended the sole use of regular insulin in sliding scale for diabetes management and about 36% did not initiate any form of treatment. Hyperglycemia in patients not known to have diabetes is less likely to be recognized.

## Conclusion

This study demonstrates the gaps in knowledge about inpatient diabetes that exist in medical school. The findings can be used to design a curriculum appropriately targeted to the level of 6th medical students.

